#  Gene expression profile associated with Asmt knockout-induced depression-like behaviors and exercise effects in mouse hypothalamus

**DOI:** 10.1042/BSR20220800

**Published:** 2022-07-14

**Authors:** Wenbin Liu, Zhuochun Huang, Jie Xia, Zhiming Cui, Lingxia Li, Zhengtang Qi, Weina Liu

**Affiliations:** 1 Key Laboratory of Adolescent Health Assessment and Exercise Intervention of Ministry of Education, East China Normal University, Shanghai 200241, China; 2 College of Physical Education and Health, East China Normal University, Shanghai 200241, China

**Keywords:** depression, differentially expressed genes (DEGs), melatonin (MT), N-acetylserotonin methyltransferase (Asmt), swimming exercise

## Abstract

Sleep disorder caused by abnormal circadian rhythm is one of the main symptoms and risk factors of depression. As a known hormone regulating circadian rhythms, melatonin (MT) is also namely N-acetyl-5-methoxytryptamine. N-acetylserotonin methyltransferase (Asmt) is the key rate-limiting enzyme of MT synthesis and has been reportedly associated with depression. Although 50–90% of patients with depression have sleep disorders, there are no effective treatment ways in the clinic. Exercise can regulate circadian rhythm and play an important role in depression treatment. In the present study, we showed that Asmt knockout induced depression-like behaviors, which were ameliorated by swimming exercise. Moreover, swimming exercise increased serum levels of MT and 5-hydroxytryptamine (5-HT) in Asmt knockout mice. In addition, the microarray data identified 10 differentially expressed genes (DEGs) in KO mice compared with WT mice and 29 DEGs in KO mice after swimming exercise. Among the DEGs, the direction and magnitude of change in epidermal growth factor receptor pathway substrate 8-like 1 (Eps8l1) and phospholipase C-β 2 (Plcb2) were confirmed by qRT-PCR partly. Subsequent bioinformatic analysis showed that these DEGs were enriched significantly in the p53 signaling pathway, long-term depression and estrogen signaling pathway. In the protein–protein interaction (PPI) networks, membrane palmitoylated protein 1 (Mpp1) and p53-induced death domain protein 1 (Pidd1) were hub genes to participate in the pathological mechanisms of depression and exercise intervention. These findings may provide new targets for the treatment of depression.

## Introduction

Sleep disorders caused by abnormal circadian rhythm have been widely demonstrated in patients with depression [1,2]. However, most of the current antidepressants have a weak therapeutic effect on sleep disorders [3], such as selective serotonin reuptake inhibitor (SSRI) and selective norepinephrine reuptake inhibitor (SNRI), which are based on the monoamine hypothesis [4]. 5-Hydroxytryptamine (5-HT), a kind of monoamine neurotransmitter, can be catalyzed by N-acetyl serotonin methyltransferase (Asmt) via N-acetyl serotonin to form melatonin (MT) [5]. Clinically, MT level decreases in patients with depression, and MT treatment can prolong sleep time and alleviate depression [6]. Animal studies have also confirmed the rapid and lasting anti-depression and anti-anxiety effects of MT in chronic stress rats [7]. Therefore, it is of great significance to explore the regulatory factors of MT secretion in depression.

In the pineal, arylalkylamine N-acetyltransferase (AANAT) and Asmt are both key rate-limiting enzymes affecting MT biosynthesis. Asmt plays a catalytic role in the last step of the synthesis reaction and has been found to be associated with depression [8]. The mRNA and protein expression levels of Asmt were significantly lower in patients with recurrent depression than those in healthy individuals [9]. It has been reported that single nucleotide polymorphism of Asmt gene is implicated in depression, the AA genotype at rs4446909 and GG genotype at rs5989681 in the promoter B region of Asmt gene were protective genes of depression [10]. Similarly, Asmt gene polymorphism has been reportedly relevant to the susceptibility of autism spectrum disorders [10] and bipolar disorders [11]. Therefore, the first aim of the present study is to identify whether Asmt knockout induces depression-like behaviors in mice, thus being employed as a useful model to study depression with sleep disorders.

Exercise has been found to regulate the phase of MT rhythm and the sleep–wake cycle [12], as well as circadian adaptation [13] by regulating MT level [14]. Moreover, exercise is an effective treatment for major depression [15]. Among the many challenges related to depression, lack of biomarkers for diagnosis appears especially prominent. Gene chip, also called DNA chip or DNA microarray, is an appropriate tool to address this issue [16]. In recent years, gene chip has been widely used in the research fields of depression [17], exercise [18], and exercise intervention for depression [19]. Therefore, the second aim of the current study is to investigate the effects of Asmt knockout and swimming exercise on gene expression profiles in mice, which may provide selective biomarkers for depression.

## Materials and methods

### Animals and groups

Mice were obtained from Humangen Biotech Inc. (Shanghai, China). Asmt-knockout mice (F0) were generated via co-injection of *in vitro*-synthesized Asmt sgRNA (5′-gCGCCTACACCAACTCCCCCC-3′), Cas9 mRNA and pMD19T-T7 vector into the zygotes of C57BL/6 mice [20]. Mouse Asmt gene (ID: 107626) is located in the F5 region of X chromosome, and a total of 5 SgRNAs were designed for exons 2 and 3, and the splicing efficiency was tested in NIH3T3 cells. The founder mice was analyzed with quantitative real-time polymerase chain reaction (qRT-PCR) using the set of primers (sense 5'-agggtcacagttcatggtgg-3' and antisense 5'-tcgacgcccacggccctcgcgta-3'). The initially obtained Asmt-knockout mice (i.e., the positive founder mice with 20 bp deletion, F0) from Humangen Biotech Inc. were backcrossed with their wild-type littermates C57BL/6 mice for three generations. Genotyping was conducted with high resolution melting (HRM) analysis after qRT-PCR, which can distinguish three genotypes accurately [21].

Mice were housed with a 12-h light:dark cycle under controlled temperature (22 ± 2°C) and humidity (50 ± 10%), and were given standard diet and water ad libitum. The breeding male mice (3- to 4-week old, 17–20 g) were performed gene identification to select Asmt-knockout homozygous mice and wild-type littermate mice. All mice were adaptively reared for 3 weeks, and were then trained in a swimming program or not. Mice were randomly assigned to four groups: wild-type control group (WT, *n*=5), Asmt-knockout control group (KO, *n*=5), wild-type exercise group (WE, *n*=4), and Asmt-knockout exercise group (KE, *n*=5). All animal work has taken place in the Experimental Animal Centre at East China Normal University, Shanghai, China.

### Exercise protocol

As described in studies of ours [22] and others [23], WE and KE mice were trained in a moderate swimming process with no weight loading in free style. Daily swimming exercise was performed in a large glass water tank [100 cm (*L*) × 60 cm (*W*) × 80 cm (*H*)] at 32 ± 1°C. The exercise period began when mice were at the age of 6–7 weeks. During the first week for adaptation, the training was graded beginning with 10 min on the first day until 60 min on the last day. Thereafter, regular training period began with intensity of 60 min/day, 6 d/week for 4 weeks. Exercise was performed at the same time every day (between 9:00 and 11:00 a.m.).

### Behavioral testing

Except sucrose preference, a videocomputerized tracking system (DigBehav, Jiliang Co. Ltd., Shanghai, China) was used to record the behavioral changes of the animals. These behavioral tests were performed as described in our previous study [24].

#### Sucrose preference test (SPT)

In the SPT, sweetness preference decreases in depressed mice [25]. Briefly, 72 h before the test mice were trained to adapt 1% sucrose solution (w/v): two bottles of 1% sucrose solution were placed in each cage, and 24 h later 1% sucrose in one bottle was replaced with tap water for 24 h. After adaptation, mice were deprived of water and food for 24 h. Thereafter, mice housed in individual cages had free access to two bottles respectively containing 200 ml of sucrose solution (1% w/v) and 200 ml of water. At the end of 24 h, the sucrose preference was calculated as a percentage of the consumed 1% sucrose solution relative to the total amount of liquid intake.

#### Forced swim test (FST)

The FST is one of the most used tools for screening antidepressants among all animal models [26]. The swimming sessions were conducted by placing the mice in cylinders (30 cm height × 10 cm diameters) containing 25°C water 20 cm deep so that the mice could not support themselves by touching the bottom with their feet. The FST was conducted for 5 min, during which immobility time and struggling time was recorded. Floating in the water without struggling and only making movements necessary to keep its head above the water were regarded as immobility.

#### Open field test (OFT)

The OFT is employed to evaluate the effects of antidepressant treatment [27]. Each mouse was placed in the center of the open field (30 × 30 × 30 cm chamber, with 16 holes in its floor, each hole had infrared sensing) for 5 min in a quiet room. The parameter assessed was the number of poking into holes.

### Sample collection

Mice were decapitated after isoflurane anesthesia and blood was kept in room temperature, and then was centrifuged at 3000 rpm for 10min to separate the serum and blood cells. The hypothalamus was rapidly and carefully separated on ice-plate, in view of regulating effects of hypothalamus on sleep, mood, circadian, and seasonal rhythm [28]. The serum and hypothalamus were stored at −80°C until assays.

### Enzyme-linked immunosorbent assay (ELISA)

Serum concentrations of MT and 5-HT were determined using commercially ELISA kits (Shanghai Enzyme-linked Biotechnology Co., Ltd., Shanghai, China) following the manufacturer’s instructions.

### Agilent transcriptome microarray assay

The collected hypothalamus tissues were put into dry ice and sent to OE Biotech Co., Ltd (Shanghai, China) for the One-Color Microarray-Based Gene Expression Detection and Analysis. Each group included three samples. The gene expression profiles were conducted using Agilent SurePrint G3 Mouse Gene Expression V2.0 (8 × 60K, Design ID: 074809).

#### Expression profile microarray experiments

Total RNA was quantified by NanoDrop ND-2000 (Thermo Scientific) and the RNA integrity was assessed using Agilent Bioanalyzer 2100 (Agilent Technologies). The RNA integrity assessment results of all samples were very good, with A (RNA Integrity Number [RIN] ≥7 and 28S/18S ≥ 0.7), which means these samples can be used in subsequent experiments. The sample labeling, microarray hybridization, and washing were performed according to the manufacturer’s standard protocols. After washing, the arrays were scanned by Agilent Scanner G2505C (Agilent Technologies) to get the array images. The raw data were extracted from the images by Feature Extraction software (version10.7.1.1, Agilent Technologies) finally.

#### Differentially expressed genes identification

Genespring (version 13.1, Agilent Technologies) was employed to process the basic analysis with the raw data. To begin with, the raw data were normalized with the quantile algorithm. The probes that at least 100% of the values in any 1 out of all conditions have flags in “Detected” were chosen for further data analysis. Differentially expressed genes (DEGs) were then identified through fold change as well as *P* value calculated with unpaired *t*-test. The screening criterion of DEGs was that the absolute fold change (FC-abs) ≥ 2.0 and *P*≤0.05. Afterward, Hierarchical Clustering (shown in the heatmap) was performed to display the differential gene expression pattern among samples by MeV 4.6 software, and the parameters were “Distance Metrics, Euclidean Distance, Linkage Method, Complete linkage”.

#### Bioinformatics analysis of expression profiles

Briefly, function annotations including biological process (BP), cellular component (CC), and molecular function (MF) were obtained based on the Gene Ontology (GO) project (http://geneontology.org/). And enriched pathways were identified using the Kyoto Encyclopedia of Genes and Genomes (KEGG) project (http://www.genome.jp/kegg/). The GO and KEGG terms were pondered significant if *P*<0.05 or 0.1.

To locate potential therapeutic targets among identified DEGs, the protein–protein interaction (PPI) networks were constructed by Search Tool for Retrieval of Interacting Genes/Proteins (STRING, version 9.0, https://string-db.org/) and Cytoscape software (version 3.2.1, http://cytoscape.org/). The minimum required interaction score was set as 0.15 in the STRING online database. Cytoscape software was used to visualize the PPI network, and the CytoHubba plugin for Cytoscape was used to calculate the degree value to screen the hub genes.

### qRT-PCR validation

The hypothalamus tissues were performed by qRT-PCR to exclude false-positive results and validate microarray assay results further. Total RNA was extracted from frozen tissues using TRIzol (Invitrogen, Chromos, Singapore) and purified according to the instructions included. Then, double-stranded cDNA was synthesized from ∼1 μg of total RNA using ReverTra Ace® qPCR RT Kit (TOYOBO, Osaka, Japan). Real-time PCR kit (TOYOBO, Osaka, Japan) was used to prepare the 20 μl reaction system, including 4 pmol of each primer, 2.0× Master SYBR Green I (contains Taq DNA polymerase, reaction buffer, dNTP mix, SYBR Green I dye, and 10 mM MgCl_2_), and 2.0 μl template. Real-time PCR reactions were cycled in QuantStudio 3 Real-Time PCR Instrument (Applied Biosystems, CA, U.S.A.). The amplification occurred in a three-step cycle (denaturation at 95°C for 15 s, annealing at 61°C for 30 s, extension and data collection at 72°C for 45 s) for 40 cycles. The target gene expression was normalized to internal standard 18S. Fold changes in the expression of genes of interest were calculated using the 2^−ΔΔCt^ method. According to mouse EST sequences of epidermal growth factor receptor pathway substrate 8-like 1 (Eps8l1), phospholipase C-β2 (Plcb2), synaptotagmin-like 2 (Sytl2), checkpoint kinase 1 (Chek1), cyclin-dependent kinase inhibitor 1A (Cdkn1a), inositol 1,4,5-triphosphate receptor 2 (Itpr2), transformed mouse 3T3 cell double minute 4 (Mdm4), p53 induced death domain protein 1 (Pidd1), heat shock protein family A member 1B (Hspa1b) and 18S in NCBI database. Primer Premier 3.0 software was used to design the primers. Primer sets used in the present study were shown in Supplementary Table S1.

### Statistical analysis

Data are presented as mean ± SEM. Analyses were performed using GraphPad Prism Software (version 6.01). The statistical significance of differences was determined using two-way ANOVA followed by Bonferroni test for post hoc comparisons. Statistical significance was set at *P*<0.05 or 0.1.

## Results

### Effects of Asmt knockout and swimming exercise on depression-like behaviors and serum levels of MT and 5-HT

**Figure 1:**
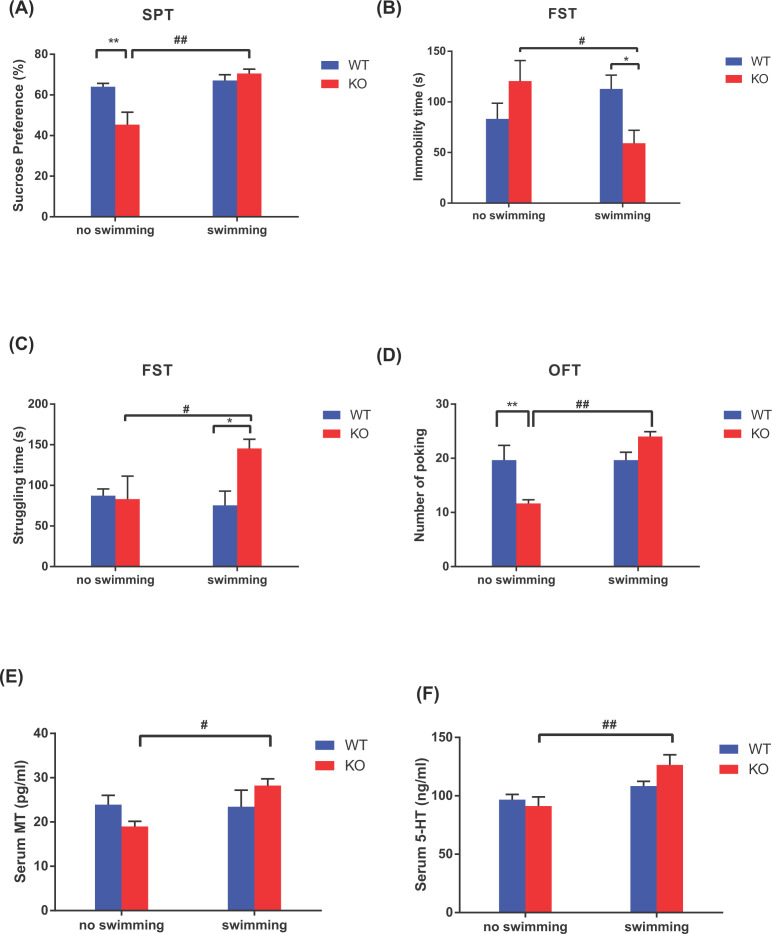
Effects of Asmt knockout and swimming exercise on depression-like behaviors and serum hormone levels in mice (**A–D**) Depression-like behaviors: (A) Sucrose preference in sucrose preference test (SPT); (B) Immobility time in forced swim test (FST); (C) Struggling time in FST, (D) Poking number in open field test (OFT); (**E** and **F**) Serum hormone levels: (E) melatonin (MT), (F) 5-hydroxytryptamine (5-HT). Data are presented as means ± SEM (*n* = 3–5 per group). *P<0.05, **P<0.01 versus WT; #*P*<0.05, ##*P*<0.01 versus no swimming.

First, we demonstrated that Asmt knockout induced depression-like behaviors, including reduced percentage of sucrose preference in SPT (*P*<0.01, Figure 1A) and poking number in OFT (*P*<0.01, Figure 1D), compared with WT group. Swimming exercise ameliorated depression-like behaviors induced by Asmt knockout, including increased percentage of sucrose preference in SPT (*P*<0.01, Figure 1A), struggling time in FST (*P*<0.05, Figure 1C) and poking number in OFT (*P*<0.01, Figure 1D), as well as decreased immobility time in FST (*P*<0.05, Figure 1B). No KO × swim interaction on behavioral testing was found.

Then, we examined the serum levels of MT and 5-HT in hypothalamus. No main effects of Asmt knockout were found on the levels of MT or 5-HT, whereas swimming exercise increased the levels of MT (*P*<0.05, Figure 1E) and 5-HT (*P*<0.01, Figure 1F). A significant KO × swim interaction was found on MT level (F_(1, 11)_ = 4.853, *P*<0.05), suggesting the association among MT, Asmt, and swim.

### Identification of DEGs after Asmt knockout and swimming exercise

**Figure 2:**
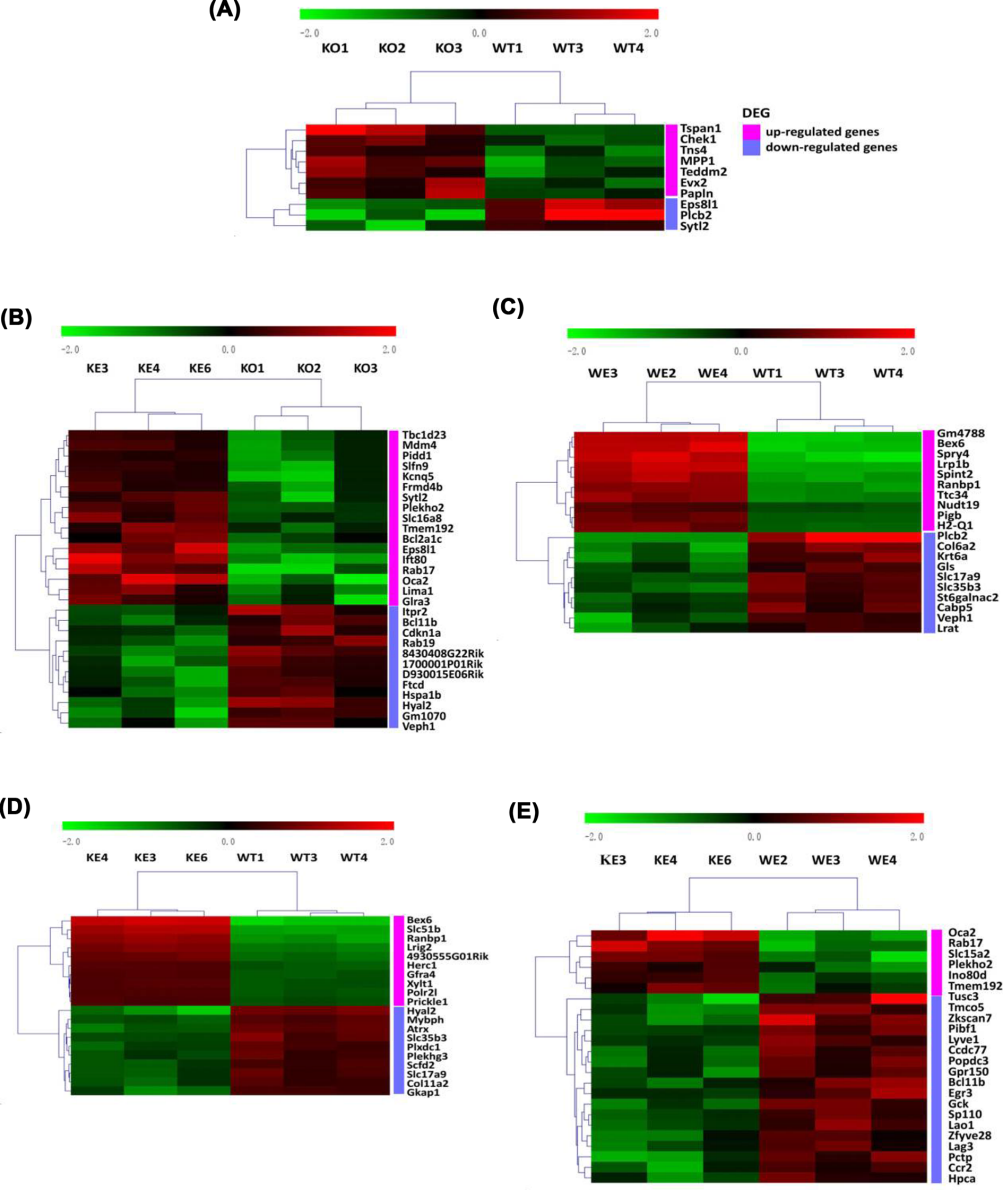
Heatmaps of the DEGs in mice hypothalamus (**A**) Asmt-knockout control group (KO) versus wild-type control group (WT), (**B**) Asmt-knockout exercise group (KE) versus KO, (**C**) wild-type exercise group (WE) versus WT, (**D**) KE versus WT, (**E**) KE versus WE. Color scale represents gene expression value from relatively low (green) to relatively high (red).

According to the strict screening criteria mentioned above, we identified a large number of DEGs, the overall results were presented in Supplementary Table S2. Of these DEGs, 7 up-regulated and 3 down-regulated genes were identified in mice hypothalamus following Asmt knockout, compared with WT group (Figure 2A and Supplementary Table 3). In addition, 17 up-regulated and 12 down-regulated genes were identified in mice hypothalamus after swimming exercise, compared to KO group (Figure 2B and Supplementary Table S4). The top 10 up-regulated and down-regulated DEGs of the other comparisons among groups were shown in Figure 2C–E, and details were listed in Supplementary Tables S5–7.

### Validation of DEGs by qRT-PCR

**Figure 3:**
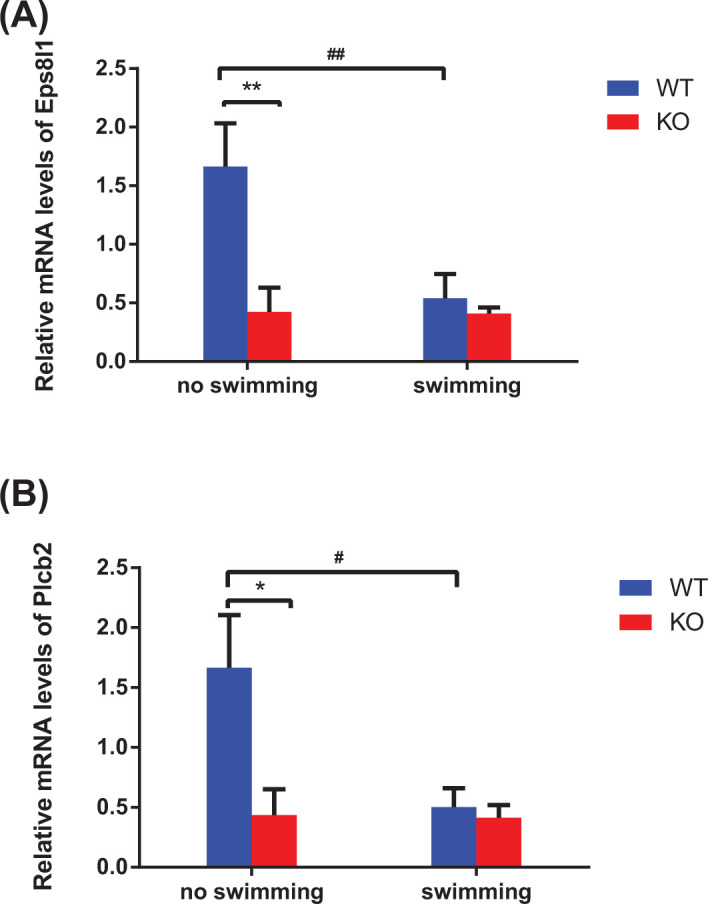
Validation of DEGs by qRT-PCR The expression levels of epidermal growth factor receptor pathway substrate 8-like 1 (Eps8l1, **A**) and phospholipase C-β 2 (Plcb2, **B**) in mice hypothalamus with response to Asmt knockout and swimming exercise. The relative mRNA levels were conducted using qRT-PCR. Data are presented as means ± SEM (*n* = 3 per group). *P<0.05, **P<0.01 versus WT; #*P*<0.05, ##*P*<0.01 versus no swimming.

To validate the microarray results, we selected nine DEGs based on *P*-value and FC-abs, the mean of expression quantity and association with Asmt or exercise, and then performed qRT-PCR. These nine DEGs included Eps8l1, Plcb2, Sytl2, Chek1, Cdkn1a, Itpr2, Mdm4, Pidd1, and Hspa1b. The qRT-PCR results of Eps8l1 (Figure 3A) and Plcb2 (Figure 3B) were consistent with the microarray data. The mRNA levels of Eps8l1 and Plcb2 were significantly down-regulated following Asmt knockout in mice, verifying the reliability of hypothalamus gene expression profiles. In view of enriched functions of Eps8l1 (Supplementary Table S8) and Plcb2 (Supplementary Table S9), we can infer that Eps8l1 and Plcb2 were related to Asmt knockout-induced depression-like behaviors. In addition, it needs to be further proved whether Eps8l1 and Plcb2 are involved in antidepressant effects of swimming exercise. The validation results of the other seven genes were shown in Supplementary Figure S1.

### Enrichment analysis of DEGs after Asmt knockout and swimming exercise

**Figure 4:**
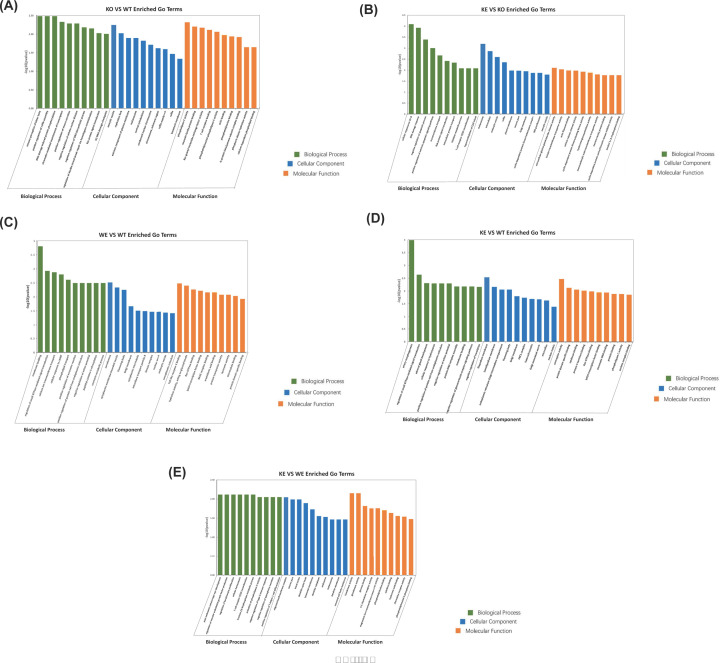
Gene Ontology (GO) analysis of DEGs (**A**) KO versus WT, (**B**) KE versus KO, (**C**) WE versus WT, (**D**) KE versus WT, and (**E**) KE versus WE. Bar charts of function enrichment analysis were constructed by -log10 (*P* value) and the top 10 GO terms. The bar color represents GO terms related to Biological Process (green), Cellular Component (blue) and Molecular Function (orange).

The function enrichment analysis of DEGs was conducted by GO analysis. Following Asmt knockout in mice, the most prominent terms of BP, CC and MF were respectively involved in “sensory perception of bitter taste” (*P*=0.003288), “exocytic vesicle” (*P*=0.005665) and “phospholipase C activity” (*P*=0.004805); Figure 4A and Supplementary Table S10. After swimming exercise in KO mice, the most prominent terms of BP, CC, and MF were respectively involved in “cellular response to UV-B” (*P*=8.09E-05), “melanosome” (*P*=0.000636) and “extracellular-glycine -gated chloride channel activity” (*P*=0.007847); Figure 4B and Supplementary Table S11. The top 10 enriched GO terms generated by the other comparisons among groups were presented in Figure 4C–E, and details were listed in Supplementary Tables S12–14.

**Figure 5:**
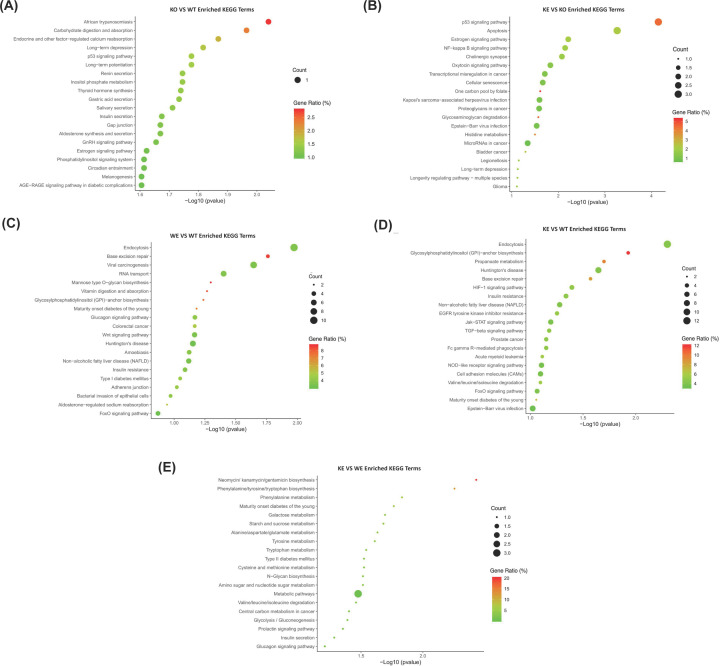
Kyoto Encyclopedia of Genes and Genomes (KEGG) pathways analysis of DEGs (**A**) KO versus WT, (**B**) KE versus KO, (**C**) WE versus WT, (**D**) KE versus WT, and (**E**) KE versus WE. Dot plots of pathway enrichment analysis were constructed by -log10 (*P* value) and the top 20 KEGG terms. The dot size represents the number of genes enriched in specific KEGG terms, and the dot color represents the gene ratio [i.e. Count (the number of genes enriched) / PopHit (the number of genes annotated)] in specific KEGG terms.

The pathway enrichment analysis of DEGs was further conducted to gain deeper insight into the effects of Asmt knockout and swimming exercise on depression-like behaviors. Based on the KEGG database, three pathways were significantly enriched following Asmt knockout and swimming exercise, such as p53 signaling pathway, long-term depression (LTD), and estrogen signaling pathway, but the involved genes were different in each comparison (Figure 5A,B and Supplementary Tables 15–17). The top 20 enriched KEGG terms generated by the other comparisons among groups were presented in Figure 5C–E, and details were listed in Supplementary Tables 18–20.

### PPI network construction of DEGs

**Figure 6:**
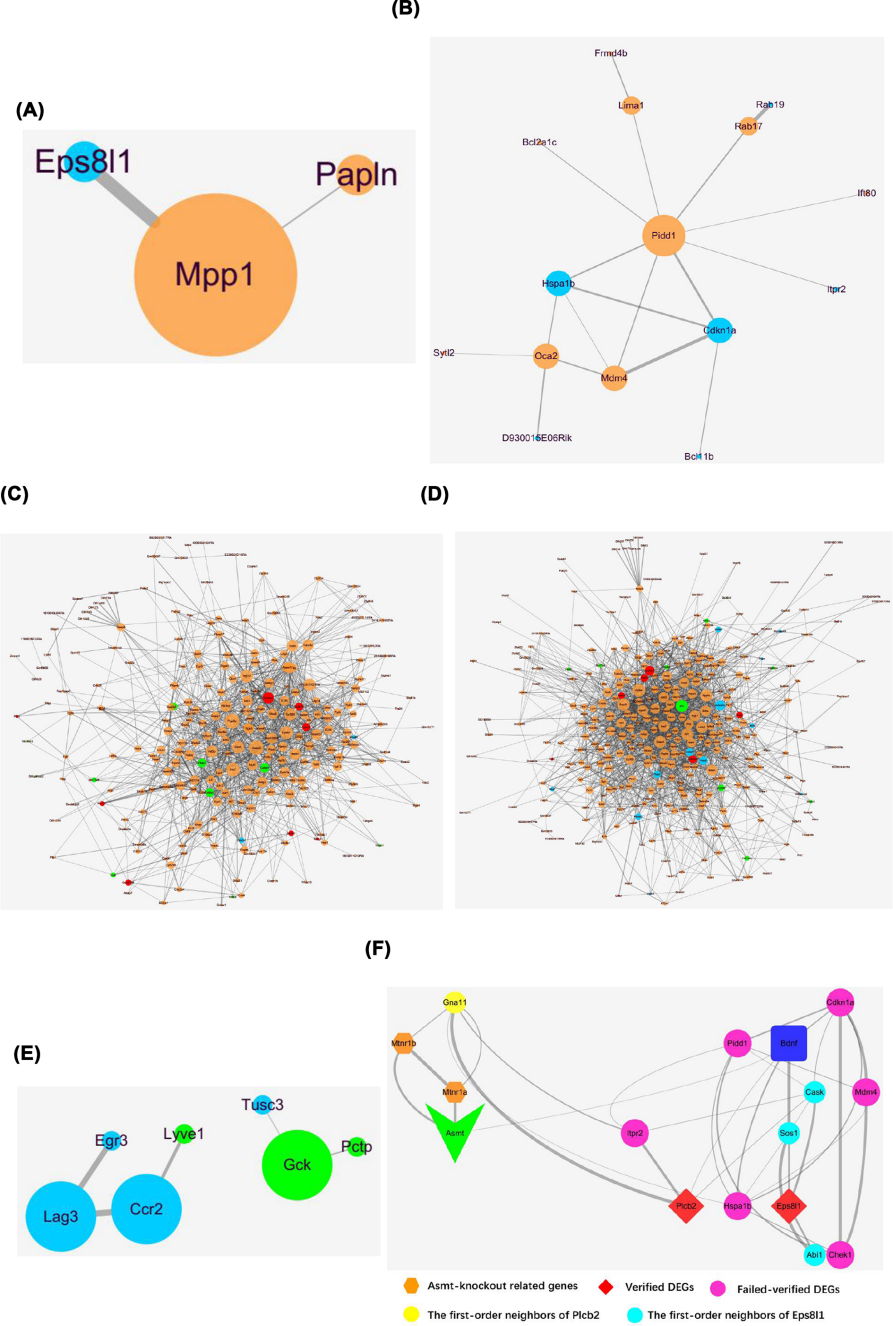
The PPI networks of DEGs (**A**) KO versus WT, (**B**) KE versus KO, (**C**) WE versus WT, (**D**) KE versus WT, and (**E**) KE versus WE. The PPI networks were constructed by the proteins represented by nodes and interactions represented by the edges between nodes. The nodes size represents the value of degree, and the edge thickness represents the intensity of interactions. Separately, orange and blue nodes represent up-regulated and down-regulated genes, with red and green nodes representing the top 10 up-regulated and down-regulated genes. (**F**) The PPI network was constructed by Asmt, MT receptors (Mtnr1a and Mtnr1b), Bdnf, verified DEGs (Eps8l1 and Plcb2), failed-verified DEGs (Chek1, Cdkn1a, Itpr2, Mdm4, Pidd1, Hspa1b), the first-order neighbors of Eps8l1 (Cask, Sos1, Abi1) and Plcb2 (Gna11). The color and shape of nodes represents different kinds of proteins, and the edge thickness represents the intensity of interactions.

Generally, a gene with a higher degree in the PPI networks is capable of causing more variation in regulation processes. A PPI network including 3 nodes and 2 edges was constructed (Figure 6A), up-regulated membrane palmitoylated protein 1 (Mpp1) (degree = 2) may be involved in the molecular mechanisms of Asmt knockout-induced depression-like behaviors. In addition, a PPI network including 15 nodes and 18 edges was constructed (Figure 6B), up-regulated Pidd1 (degree = 8) may be involved in the regulation mechanisms of swimming exercise for depression-like behaviors. The PPI networks of the other comparisons among groups were shown in Figure 6C–E.

In order to further investigate the roles of identified DEGs in the development of depression, then we constructed another network (Figure 6F) among Asmt, brain-derived neurotrophic factor (Bdnf), MT receptors (Mtnr1a and Mtnr1b), identified DEGs (Eps8l1, Plcb2, Chek1, Cdkn1a, Itpr2, Mdm4, Pidd1, Hspa1b), the first-order neighbors of Eps8l1 (Cask, Sos1, Abi1) and Plcb2 (Gna11). As shown in Figure 6F, Asmt may be participated in the molecular mechanism of depression-like behaviors and swimming effects through Bdnf, then Asmt knockout can lead to abnormal expression of Ep8l1 via Sos1. Probably, Asmt knockout play an important role through Mtnr1b/Gna11/Plcb2/Bdnf on depression-like behaviors. Although other identified DEGs from microarray data were not successfully verified by PCR, they may be associated with the pathogenesis of depression and regulation of exercise according to this network.

## Discussion

To our knowledge, this is the first study to report the animal model of depression induced by Asmt knockout. Of course, it needs further study to confirm the model validity. Initially, we aimed to build Asmt knockout mice to explore the role of MT in depression. However, we ignored the congenital deficiency of Asmt in C57BL/6J mice during gene targeting, thus constructing a mouse strain that seemed to make little sense. Then we thought this question deeply from a different perspective, mouse strain and genotype cannot be ignored when treated with antidepressant therapy [29–31]. Even if C57BL/6J mice were born with melatonin deficiency, genetic deletion of MT1 melatonin receptor caused depressive and anxiety-like behaviors in male and female C57BL/6 mice [32]. In fact, the comparison of different strains of animals showed that the behaviors of C57BL/6J mice in FST, TST, and OFT were similar to other strains [33].

Asmt is the key rate-limiting enzyme of MT [5]. MT has positive effects on depression by regulating neurogenesis [34], neuroplasticity [35], and neurotransmitters [36]. More importantly, MT can improve depression accompanied with anxiety and sleep disorder through oxidative stress and immune response [37]. Therefore, we suppose that Asmt knockout-induced depression-like behaviors may be associated with MT expression. However, Asmt knockout did not significantly affect melatonin levels in our study. Consistently, C57BL/6J mouse strain, which was often used to construct animal models of depression, are inborn with lower Asmt activity in the previous studies. Interestingly, we found that the KE animals show better improvement than WE animals in the behavioral studies, including reduced immobility time in FST (*P*<0.01, Figure 1B) and increased struggling time in FST (*P*<0.01, Figure 1C). It is suggested that male mice with Asmt knockout have an easier time reaping the benefits of exercise than wild-type. The possible explanation for this is that Asmt knockout strengthens neurobehavioral response to exercise due to a significant KO × swim interaction on the MT level. Furthermore, this may be related to a day-night difference in the response of MT level to swimming [38]. As the substrate of MT formation catalyzed by Asmt, 5-HT is a critical factor linking dysregulation of circadian system and mood [39]. Aerobic exercise has been found to enhance activities of brain serotonergic projections [40]. In the current study, we also found that swimming exercise increased serum 5-HT (i.e. serotonin) level.

To dissect the molecular and genetic basis underlying the association among depression-like behaviors, Asmt knockout and swimming exercise, we have used transcriptome microarray technology. 10 DEGs were identified in mice following Asmt knockout compared with WT group, and 29 DEGs were identified in mice after swimming exercise compared to KO group. Nine DEGs were selected to validate the microarray results, whereas only Eps8l1 and Plcb2 were verified by qRT-PCR. Eps8 has three gene analogs, Eps8l1, Eps8l2, and Eps8l3 [41,42]. Our results showed that Eps8l1 mRNA level in KO group was significantly down-regulated compared to WT group. Similarly, the down-regulation of Eps8 was accompanied by negative effects on cell proliferation, differentiation, and survival [43]. On the contrary, Eps8 has been reported to be up-regulated in most cancer patients, including pituitary gland cancer, breast cancer, ovarian cancer, and so on [44]. Cancer patients have been reported a higher risk of depression [45]. The inconsistency may be due to expression trend in depression and cancer subjects, and also due to expression differences of Eps8 and three analogs. It has been reported that Eps8l1 and Eps8l2, but not Eps8l3, restore receptor tyrosine kinase-dependent actin remodeling and link growth factor stimulation to actin organization [42]. The regulatory effects of Eps8l1 on actin dynamics suggested that it may be connected with exercise intervention for diseases. Consistently, Eps8l1 was one of up-regulated genes after swimming exercise in microarray results. Furthermore, GO terms analysis showed that Eps8l1 was involved in Rac guanyl-nucleotide exchange factor activity, which has been found to regulate glucose metabolism in brain and then relate to brain diseases [46].

Besides, Eps8l1 is crucial for the Eps8-Abi1-Sos1 complex that required for Rac activation leading to actin cytoskeletal remodeling [46]. Sos1 is a guanine nucleotide exchange factor, and Abi1 acts as a scaffold between Eps8 and Sos1 assemble [47]. Cask, another first-order neighbor of Eps8l1, belongs to the membrane-associated guanylate kinase (MAGUK) family, and improve neurodevelopmental disorders by regulating synaptic plasticity [48,49]. These findings suggested that Eps8l1 may be concerned with brain diseases and exercise effects. According to the PPI network (Figure 6F), the effects of Asmt knockout and swimming exercise may be connected with Bdnf, resulting in down-regulation of Eps8l1 via Sos1. Bdnf is important in improving depression-like behaviors by stabilizing hippocampal synaptic plasticity, thus it is the most sensitive neurotrophic factor in antidepressant effects of exercise [50]. Overall, Eps8l1 may take part in molecular mechanism of depression pathology and exercise effects via regulating synaptic plasticity and actin structures.

Plcb2 has been found to mainly distribute in oligodendrocytes and modulate the transmission of neuroendocrine signals [51]. Plcb2 binding to Gna11 is responsible for the regulation of phospholipase C (PLC) in diverse systems [52]. PLC is the key enzyme of phosphatidylinositol signaling pathway, which is closely related to neuron activity and exercise effect. It has been reported that PLC signal disorder is involved in neuropsychiatric diseases including depression, bipolar disorder, schizophrenia, Huntington’s disease, and Alzheimer’s disease [51]. Six subtypes of PLC have been identified in mammals, namely PLC β, PLC γ, PLC δ, PLC ε, PLC ζ and PLC η. Among them, PLC β is encoded by the Plcb gene, including Plcb1, Plcb2, Plcb3, and Plcb4. Plcb1-related signal pathway related to depression [53], and chronic treatment with quetiapine changed Plcb1 mRNA level from microarray analysis [54]. Our results of enrichment analysis suggested that Plcb2 may be also one of depression-related target molecules like Plcb1. GO terms enriched by Plcb2 mainly included sensory perception of bitter taste, cytoplasm, PLC activity, etc. KEGG terms enriched by Plcb2 included LTD, estrogen signaling pathways, circadian entrainment, melanogenesis, carbohydrate digestion and absorption, and AGE-RAGE signaling pathway in diabetic complications. Our results showed that Plcb2 mRNA level was reduced following Asmt knockout in mice. Consistent with the PPI network (Figure 6F), Asmt knockout may play an important role through Mtnr1b/Gna11/Plcb2/Bdnf in depression. Taken together, Plcb2 may be a representative molecular marker of depression through the effects on circadian rhythm and metabolism, which are crucial for depression [55] and exercise [56].

It is worth noting that MT levels in Asmt KO mice decreased compared with WT mice, but there was no significant difference. If the MT levels were not altered in Asmt KO mice, Eps8l1 and Plcb2 might ameliorate depressive symptoms in a more direct way. After Asmt was knocked out, the mRNA expression levels of Eps8l1 and Plcb2 decreased (Figures 2A,B and 3A,B). Then Eps8l1 can down-regulate Bdnf via Sos1, and Plcb2 can down-regulate Bdnf directly (Figure 6F). This reasonable conjecture is supported by evidence from the previous study. It is reported that Eps8 regulates axonal filopodia in hippocampal neurons in response to Bdnf, this is a process with crucial impacts on neuronal development and synapse formation [57]. The results indicated that 5-HT affected Bdnf secretion from NG2 cells via the PLC signaling pathway [58]. And the cortical oligodendrocytes regulated the release of Bdnf through the PLC pathway [59]. In other words, Eps8l1 and Plcb2 may regulate synaptic plasticity by affecting the expression level of Bdnf to participate in the molecular mechanisms of depression.

In addition, our microarray data show that the RIKEN cDNA 8430408G22 gene, RIKEN cDNA D930015E06 gene, and RIKEN cDNA 1700001P01 gene down-regulated significantly in the antidepressant effects of exercise (Supplementary Table S4). Long non-coding RNA (LncRNA) is defined as a non-coding RNA molecule with a length of more than 200 nucleotides and was initially described by Okazaki et al in a large-scale sequencing study of a full-length mouse cDNA library in 2002. Recently, the roles of lncRNA in the pathogenesis of depression have been shown to regulate synaptic plasticity, Bdnf expression, inflammation and so on [60]. LncRNA networks are differentially modified during endurance exercise in mice with depression-like behaviors [61]. Further, it was confirmed that Baduanjin can effectively ameliorate the symptoms of depression in patients with depression by regulating the dysregulated expression of lncRNA, mRNA, and circRNA [62]. So far, the researchers have raised some predictions of lncRNA function, and their signal pathway and related regulatory networks still need to be further explored in the experiments.

KEGG analysis also revealed that p53 signaling pathway, LTD, and estrogen signaling pathway were enriched significantly following Asmt knockout and swimming exercise. p53 signaling pathway is extremely essential for oxidative stress imposed by exercise [63] and brain development [64,65]. p53 dysfunction was involved in depression and medicating apoptosis [66]. Recently, p53 loss has been found to drive neuron reprogramming in cancer [67]. Consistently, Eps8l1 is also a key molecule in cancer research. These results suggest that there may be a comorbidity between cancer and depression induced by Asmt knockout. LTD pathway is also a kind of synapse plasticity form depending on the change of postsynaptic Ca^2^^+^ [68]. The increased LTD has been reported to be reversed by antidepressants in rat hippocampal CA1 region [69]. The synaptic plasticity of cortical striatum was involved in learning and exercise control [70]. Additionally, estrogen signaling pathway related to postpartum depression [71]. Women are 2.5 times more likely to suffer from major depressive disorder (MDD) than men, which may due to the reduced estrogen level [72]. Depression is a kind of abnormal disease of brain energy metabolism, the estradiol combining with estrogen receptors can modulate emotion and energy homeostasis through c-fos neural activity in amygdala and hypothalamus [73]. Further study is needed to explore the roles of p53 signaling pathway, LTD and estrogen signaling pathway in Asmt-related depression and exercise effects.

Finally, we identified two hub genes as candidate therapeutic target genes, Mpp1 and Pidd1, which play important roles in PPI networks. Following Asmt knockout in mice, Mpp1 is the core gene in the PPI network. Mpp1 has been reported to regulate neutrophil polarization by AKT1 phosphorylation [74]. Depression can be regarded as an inflammatory disorder, whose pathophysiological mechanism is associated with AKT activity [75]. Mpp1, a skeleton protein in the erythrocyte membrane, belongs to the MAGUK family [76]. Based on the above statements, Mpp1 may be an important target molecule related to depression and exercise effects. After swimming exercise in KO mice, Pidd1 is the core gene in the PPI network. It is reported that Pidd1 may be participated in depression pathogenesis via Bdnf in the downstream [77]. KEGG terms enriched by Pidd1 included negative regulation of p53 signaling pathway, apoptosis, NF-kappaB signaling pathway, etc. The previous study has found that Pidd1 connected p53/TP53 to apoptosis as a component of DNA damage/stress response pathway [78]. Pidd1 is also important in the activation of NF-kappaB [79]. It has been proposed that the antagonistic relationship between p53 and NF-kappaB may explain the conversion between mania and depression state in bipolar disorders patients [80]. Moreover, exercise effects have been widely studied in regulating NF-kappaB and improving depression.

Asmt knockout induced depression-like behaviors, which were ameliorated by swimming exercise. Our results predicted some meaningful target genes as the biomarkers of depression-like behaviors and swimming exercise. Of these, Eps8l1 and Plcb2 were the first time to be proposed as the target genes in depression. Moreover, they may also be regulated by exercise. In addition, our bioinformatic results showed that p53 signaling pathway, long-term depression and estrogen signaling pathway were enriched significantly. As the hub genes in the PPI networks, the expression of Mpp1 and Pidd1 may provide diagnostic and therapeutic value for depression. Deficiently, the small sample size and differences of RNA extraction methods in different laboratories may affect the results in the present study. In addition, circadian rhythm were not recorded in mice. Consequently, further study is needed to understand the underlying mechanism of these DEGs (including Eps8l1, Plcb2, Mpp1, Pidd1) in Asmt-related depression and exercise effects, thus provide new targets for the treatment of exercise for depression.

In summary, many laboratory mice, including C57BL/6J, are born with lower Asmt activity and MT deficiency due to the mutations at the Asmt. Thus, the idea that MT deficiency causes depression may be not reasonable in C57BL/6J mice. But it may affect the body’s response to exercise, which can play an important role in depression treatment. Our results suggest that Asmt and exercise may affect the depression neurobehavior responsiveness in male C57BL/6J mice. Also, the present study concludes that Asmt knockout increase neurobehavioral response to exercise, probably due to changed related gene expression levels. These findings may provide new targets for the treatment of exercise for depression.

## Supplementary Material

Supplementary Figure S1 and Tables S1-S20

## Data Availability

The microarray dataset is available at the National Center for Biotechnology Information’s Gene Expression Omnibus database (GEO accession: GSE197888).

## Abbreviations

5-HT: 5-hydroxytryptamine
Asmt: N-acetyl serotonin methyltransferase
Bdnf: brain-derived neurotrophic factor
BP: biological process
CC: cellular component
Cdkn1a: cyclin-dependent kinase inhibitor 1A
Chek1: checkpoint kinase 1
DEGs: differentially expressed genes
ELISA: enzyme-linked immunosorbent assay
Eps8l1: epidermal growth factor receptor pathway substrate 8-like 1
FST: forced swim test
GO: Gene Ontology
Hspa1b: heat shock protein family A member1B
Itpr2: inositol 1,4,5-triphosphate receptor 2
KEGG: Kyoto Encyclopedia of Genes and Genomes
LTD: long-term depression
Mdm4: transformed mouse 3T3 cell double minute 4
MF: molecular function
Mpp1: membrane palmitoylated protein 1
MT: melatonin
OFT: open field test
Pidd1: p53-induced death domain protein 1
PLC: phospholipase C
Plcb2: phospholipase C-β 2
PPI: protein–protein interaction
qRT-PCR: quantitative real-time polymerase chain reaction
SPT: sucrose preference test
Sytl2: synaptotagmin-like 2
