# Supplementary material for: Gene expression profile associated with Asmt knockout-induced depression-like behaviors and exercise effects in mouse hypothalamus
Source: Biosci Rep. 2022 Jul 14;42(7):BSR20220800. doi: 10.1042/BSR20220800 (PMC9284346; doi:10.1042/BSR20220800)
Supplement: Supplementary Figure S1 and Tables S1-S20 [file BSR-2022-0800_supp.pdf]

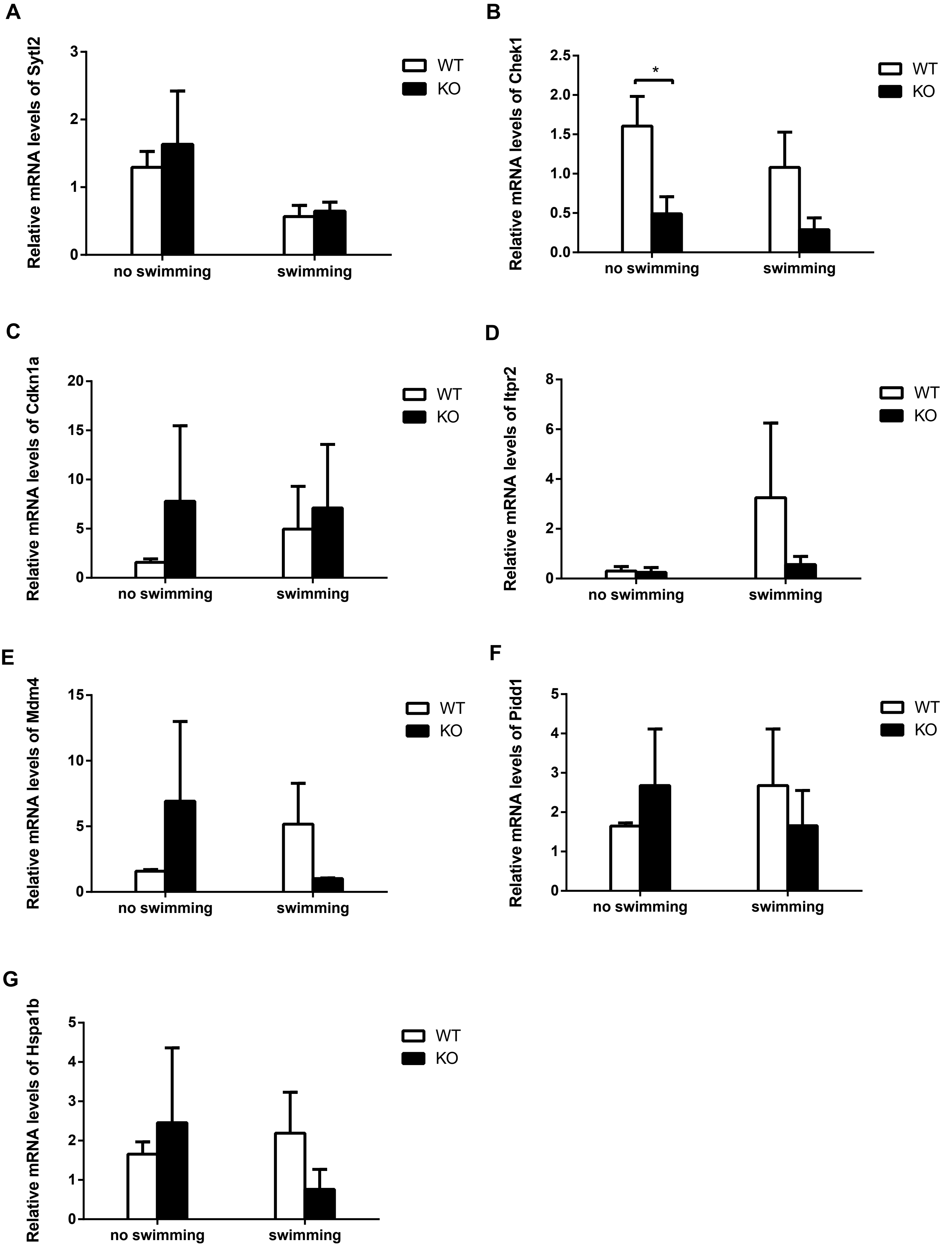
A
Relative mRNA levels of Sytl2
3
2
1
0
no swimming
swimming
WT
KO
B
Relative mRNA levels of Chek1
2.5
2.0
1.5
1.0
0.5
0.0
*
no swimming
swimming
WT
KO
C
Relative mRNA levels of Cdkn1a
20
15
10
5
0
no swimming
swimming
WT
KO
D
Relative mRNA levels of Itpr2
8
6
4
2
0
no swimming
swimming
WT
KO
E
Relative mRNA levels of Mdm4
15
10
5
0
no swimming
swimming
WT
KO
F
Relative mRNA levels of Pidd1
5
4
3
2
1
0
no swimming
swimming
WT
KO
G
Relative mRNA levels of Hspa1b
5
4
3
2
1
0
no swimming
swimming
WT
KO

Figure S1

Figure S1. Validation of DEGs by qRT-PCR. The expression levels of the other seven genes in hypothalamus in response to Asmt knockout and swimming exercise. (A) synaptotagmin-like 2 (Styl2), (B) checkpoint kinase 1 (Chek1), (C) cyclin-dependent kinase inhibitor 1a (Cdkn1a), (D) inositol 1,4,5-triphosphate receptor 2 (Itpr2), (E) transformed mouse 3T3 cell double minute 4 (Mdm4), (F) p53 induced death domain protein 1 (Pidd1), and (G) heat shock protein 1b (Hspa1b). The relative mRNA levels was conducted using qRT-PCR. Data are presented as means ± SEM (n = 3 per group). * $p < 0.05$ versus WT.

Supplementary Table 1. Gene-specific primers for quantitative real-time PCR.

Primer name	Sequence
Eps8l1	F: 5’-TCATTTGATCCCGGCTCGAT-3’<br>R: 5’-TCCCTTTCCCCATTCCCATC-3’
Plcb2	F: 5’-CTCTTCTCCTGTCCCCTGG-3’<br>R: 5’-TCTGCAAAGCTCCCAAGC-3’
Sytl2	F: 5’-GGCCTAACCATCCACGAGAG-3’<br>R: 5’-GAATCGCTTGGGCTTGACAC-3’
Chek1	F: 5’-GAGAAAGGGAATTCTGAGGTG-3’<br>R: 5’-TGGTGGGTTTAATGTCCAGC-3’
Cdkn1a	F: 5’-GGTGGGCTTATCTGGGATGG-3’<br>R: 5’-AAACAGGGATGTTTGGGGCT-3’
Itpr2	F: 5’-GGAAGAGAGGCCTGTCATGT-3’<br>R: 5’-GTGATGCTGCCATTCTCCAG-3’
Mdm4	F: 5’-GATCCTCCTGCCTGGCTTAC-3’<br>R: 5’-CACAGACAGTGCATGCAGAC-3’
Pidd1	F: 5’-CTTTGTCCCTCCACGAGTCC-3’<br>R: 5’-CCTGAGGAGCAGAGAAGTGC-3’
Hspa1b	F: 5’-CTGCTTGGGCACCGATTACT-3’<br>R: 5’-TCCCAGGCTACTGGAACACT-3’
18S	F: 5’-GTAACCCGTTGAACCCCATT-3’<br>R: 5’-CCATCCAATCGGTAGTAGCG-3’

Supplementary Table 2. The overall results of DEGs.

Comparison	Up-regulated DEGs	Down-regulated DEGs	Total
KO VS WT	7	3	10
KE VS KO	17	12	29
WE VS WT	359	17	376
KE VS WT	455	29	484
KE VS WE	6	18	24

Supplementary Table 3. The DEGs between the KO and WT groups.

Gene symbol	Gene name	*P* value	Fold change	RefSeq gene name
**Up-regulated genes**				
Mpp1	membrane palmitoylated protein 1	0.00806796	3.17	AK165261
Tspan1	tetraspanin 1	0.00955007	3.80	NM_133681
Chek1	checkpoint kinase 1	0.01303070	2.13	NM_007691
Tns4	tensin 4	0.01873418	2.06	/
Evx2	even skipped homeotic gene 2 homolog	0.03593857	2.18	NM_007967
Teddm2	transmembrane epididymal family member 2	0.03639276	2.31	NM_178243
Papln	proteoglycan-like sulfated glycoprotein	0.04980407	2.11	NM_001205343
**Down-regulated genes**				
Eps8l1	EPS8-like 1	0.00422430	-3.77	NM_026146
Plcb2	phospholipase C, beta 2	0.01100710	-8.11	NM_177568
Sytl2	synaptotagmin-like 2	0.03631679	-2.35	NR_110348

Supplementary Table 4. The DEGs between the KE and KO groups.

Gene symbol	Gene name	*P* value	Fold change	RefSeq gene name
**Up-regulated genes**				
Ift80	intraflagellar transport 80	0.00142910	5.60	NM_026641
Plekho2	pleckstrin homology domain containing, family O member 2	0.00238690	2.15	AK172714
Eps8l1	EPS8-like 1	0.00304345	3.97	NM_026146
Oca2	oculocutaneous albinism II	0.00556511	5.86	NM_021879
Rab17	RAB17, member RAS oncogene family	0.00856209	4.51	NM_008998
Slc16a8	solute carrier family 16 (monocarboxylic acid transporters), member 8	0.01601820	2.01	NM_020516
Lima1	LIM domain and actin binding 1	0.01671470	2.62	AK028311
Tbc1d23	TBC1 domain family, member 23	0.02440666	2.01	AK028852
Tmem192	transmembrane protein 192	0.02603117	2.16	AK090347
Mdm4	transformed mouse 3T3 cell double minute 4	0.02605572	2.18	AK028493
Slfn9	schlafen 9	0.02928562	2.36	NM_172796
Pidd1	p53 induced death domain protein 1	0.03002435	2.04	XM_006536220
Frmd4b	FERM domain containing 4B	0.03248306	2.09	AK051779
Kcnq5	potassium voltage-gated channel, subfamily Q, member 5	0.03835360	2.55	/
Sytl2	synaptotagmin-like 2	0.03854868	2.45	NR_110348
Bcl2a1c	B cell leukemia/lymphoma 2 related protein A1c	0.04754736	2.03	NM_007535
Glra3	glycine receptor, alpha 3 subunit	0.04915356	2.59	AK038618
**Down-regulated genes**				
8430408G22Rik	RIKEN cDNA 8430408G22 gene	0.00433508	-2.71	NM_145980
Hyal2	hyaluronoglucosaminidase 2	0.00851087	-3.08	AK087358
Gm1070	predicted gene 1070	0.01415500	-2.72	XR_105056
Bcl11b	B cell leukemia/lymphoma 11B	0.01569456	-2.09	XM_006516114
D930015E06Rik	RIKEN cDNA D930015E06 gene	0.02218209	-2.27	NM_172681
Rab19	RAB19, member RAS oncogene family	0.02307621	-2.29	NM_011226
1700001P01Rik	RIKEN cDNA 1700001P01 gene	0.02428712	-2.30	NM_028156
Ftcd	formiminotransferase cyclodeaminase	0.03222452	-2.18	NM_080845
Itpr2	inositol 1,4,5-triphosphate receptor 2	0.03246053	-2.31	/
Cdkn1a	cyclin-dependent kinase inhibitor 1A (P21)	0.03963165	-2.07	NM_007669
Hspa1b	heat shock protein 1B	0.04467700	-2.03	NM_010478
Veph1	ventricular zone expressed PH domain-containing 1	0.04984868	-2.14	NM_145820

Supplementary Table 5. The top 10 DEGs between the WE and WT groups.

Gene symbol	Gene name	*P* value	Fold change	RefSeq gene name
**Up-regulated genes**				
Gm4788	predicted gene 4788	0.00000243	7.46	NM_001029977
Lrp1b	low density lipoprotein-related protein 1B	0.00000593	6.59	XM_006498459
Ranbp1	RAN binding protein 1	0.00000763	4.28	AK151167
Bex6	brain expressed gene 6	0.00001760	8.10	NM_001033539
Pigb	phosphatidylinositol glycan anchor biosynthesis, class B	0.00001830	2.67	AK161911
Spry4	sprouty homolog 4	0.00002970	8.60	NM_011898
Nudt19	nucleoside diphosphate linked moiety X-type motif 19	0.00003460	2.16	NM_033080
Ttc34	tetratricopeptide repeat domain 34	0. 00003540	4. 03	XM_006538862
Spint2	hepatocyte growth factor activator inhibitor type 2 (Hai2)	0. 00004560	5. 34	AF099016
H2-Q1	histocompatibility 2, Q region locus 1	0. 00005890	2. 96	NM_010390
**Down-regulated genes**				
Slc17a9	solute carrier family 17, member 9	0.00078700	-2.29	BC019537
Krt6a	keratin 6A	0.00364642	-3.32	NM_008476
St6galnac 2	ST6(alpha-N-acetyl-neuraminyl-2,3-beta-galactosyl-1,3)-N-acetylgalactosaminide alpha-2,6-sialyltransferase 2	0.00383534	-2.04	NM_009180
Slc35b3	solute carrier family 35, member B3	0.00445461	-2.48	AK167051
Plcb2	phospholipase C, beta 2	0.00472553	-10.15	NM_177568
Col6a2	collagen, type VI, alpha 2	0.00527168	-2.98	AK044870
Gls	glutaminase	0.01436419	-2.03	AK046297
Cabp5	calcium binding protein 5	0.01656183	-2.08	NM_013877
Veph1	ventricular zone expressed PH domain-containing 1	0.02253773	-2.37	NM_145820
Lrat	lecithin-retinol acyltransferase (phosphatidylcholine-retinol-O-acyltransferase)	0.03409176	-2.03	NM_023624

Supplementary Table 6. The top 10 DEGs between the KE and WT groups.

Gene symbol	Gene name	*P* value	Fold change	RefSeq gene name
**Up-regulated genes**				
Slc51b	solute carrier family 51, beta subunit	0.00000138	5.70	NM_178933
Bex6	brain expressed gene 6	0.00000160	8.47	NM_001033539
Gfra4	glial cell line derived neurotrophic factor family receptor alpha 4	0.00000314	2.28	NM_001271002
Polr2l	polymerase (RNA) II (DNA directed) polypeptide L	0.00000422	2.23	NM_025593
Lrig2	leucine-rich repeats and immunoglobulin-like domains 2	0.00000533	3.55	AK158022
Xylt1	xylosyltransferase 1	0.00000637	2.27	NM_175645
Herc1	hect (homologous to the E6-AP (UBE3A) carboxyl terminus) domain and RCC1 (CHC1)-like domain (RLD) 1	0.00000649	2.38	AK035553
Ranbp1	RAN binding protein 1	0.00000698	4.67	AK151167
4930555G01Rik	RIKEN cDNA 4930555G01 gene	0.00000956	2.96	NM_175393
Prickle1	prickle homolog 1 (Drosophila)	0.00001030	2.15	NM_001033217
**Down-regulated genes**				
Mybph	myosin binding protein H	0.00014600	−2.32	NM_016749
Atrx	alpha thalassemia/mental retardation syndrome X-linked homolog	0.00025200	−2.65	AK172134
Scfd2	sec1 family domain containing 2	0.00079800	−2.02	AK044408
Hyal2	hyaluronidase 2	0.00108229	−3.93	AK087358
Slc35b3	solute carrier family 35, member B3	0.00144001	−2.35	AK167051
Plxdc1	plexin domain containing 1	0.00183679	−2.11	AK040620
Slc17a9	solute carrier family 17, member 9	0.00288047	−2.09	BC019537
Plekhg3	pleckstrin homology domain containing, family G	0.00312320	−2.00	AK080928
Col11a2	collagen, type XI, alpha 2	0.00315107	−2.01	NM_009926
Gkap1	G kinase anchoring protein 1	0.00341408	−2.27	XM_011244543

Supplementary Table 7. The DEGs between the KE and WE groups.

Gene symbol	Gene name	*P* value	Fold change	RefSeq gene name
**Up-regulated genes**				
Oca2	oculocutaneous albinism II	0.002111519	5.74	NM_021879
Ino80d	INO80 complex subunit D	0.003547891	2.01	AK086434
Rab17	RAB17, member RAS oncogene family	0.004100437	4.23	NM_008998
Slc15a2	solute carrier family 15 (H+/peptide transporter), member 2	0.005312482	3.56	NM_021301
Plekho2	pleckstrin homology domain containing, family O member 2	0.022072747	2.16	AK172714
Tmem192	transmembrane protein 192	0.03725582	2.04	AK090347
**Down-regulated genes**				
Pibf1	progesterone immunomodulatory binding factor 1	0.004009988	−2.18	AK030385
Gck	glucokinase	0.006009245	−2.56	NM_001287386
Tmco5	transmembrane and coiled-coil domains 5	0.007638294	−2.92	NM_026104
Zkscan7	zinc finger with KRAB and SCAN domains 7	0.009346905	−3.61	NM_001177505
Sp110	Sp110 nuclear body protein	0.009538312	−2.00	NM_175397
Lyve1	lymphatic vessel endothelial hyaluronan receptor 1	0.011785862	−2.01	NM_053247
Ccdc77	coiled-coil domain containing 77	0.013087017	−2.34	NM_026028
Lao1	L-amino acid oxidase 1	0.013674419	−2.23	NM_133892
Popdc3	popeye domain containing 3	0.014859548	−2.36	NM_024286
Pctp	phosphatidylcholine transfer protein	0.015091728	−2.99	BC053478
Bcl11b	B cell leukemia/lymphoma 11B	0.021204049	−2.43	XM_006516114
Gpr150	G protein-coupled receptor 150	0.023718348	−2.17	NM_175495
Egr3	early growth response 3	0.026980145	−2.05	NM_001289925
Zfyve28	zinc finger, FYVE domain containing 28	0.034588687	−2.09	NM_001015039
Lag3	lymphocyte-activation gene 3	0.03522037	−2.04	NM_008479
Tusc3	tumor suppressor candidate 3	0.035297345	−3.98	AK168664
Ccr2	chemokine (C-C motif) receptor 2	0.036493722	−2.17	NM_009915
Hpca	hippocalcin	0.038588762	−2.02	BU504979

Supplementary Table 8. GO terms significantly enriched by Eps8l1 in the comparison of KO versus WT and KE versus KO groups.

ID	GO term	Regulation	
		***P* value (KO VS WT)**	***P* value (KE VS KO)**
	**Biological Process**		
GO:1900029	positive regulation of ruffle assembly	0.003287926	0.009530627
GO:0016601	Rac protein signal transduction	0.009368012	0.026997436
	**Cellular Component**		
GO:0001726	ruffle	0.034013467	0.004371725
	**Molecular Function**		
GO:0030676	Rac guanyl-nucleotide exchange factor activity	0.006721637	0.018217986
GO:0042608	T cell receptor binding	0.007678511	0.020794221

Supplementary Table 9. GO terms significantly enriched by Plcb2 between the KO and WT groups.

ID	GO term	Regulation *P* value (KO VS WT)
	**Biological Process**	
GO:0050913	sensory perception of bitter taste	0.003287926
GO:0001580	detection of chemical stimulus involved in sensory perception of bitter taste	0.014950453
GO:0016042	lipid catabolic process	0.046498252
	**Cellular Component**	
GO:0005737	cytoplasm	0.047224936
GO:0005829	cytosol	0.489489852
	**Molecular Function**	
GO:0004629	phospholipase C activity	0.004805373
GO:0004435	phosphatidylinositol phospholipase C activity	0.008634546
GO:0031683	G-protein beta/gamma-subunit complex binding	0.01197408
GO:0008081	phosphoric diester hydrolase activity	0.02240329
GO:0005509	calcium ion binding	0.028870235
GO:0005543	phospholipid binding	0.039253587

Supplementary Table 10. The top 10 enriched GO terms of DEGs between the KO and WT groups.

ID	GO term	Enriched genes	*P* value
	**Biological Process**		
GO:0050913	sensory perception of bitter taste	Plcb2	0.003287926
GO:1900029	positive regulation of ruffle assembly	Eps8l1	0.003287926
GO:0006975	DNA damage induced protein phosphorylation	Chek1	0.003287926
GO:0048096	chromatin-mediated maintenance of transcription	Chek1	0.004694026
GO:0070257	positive regulation of mucus secretion	Sytl2	0.005162325
GO:0045839	negative regulation of mitotic nuclear division	Chek1	0.005162325
GO:2000279	negative regulation of DNA biosynthetic process	Chek1	0.006566021
GO:0010569	regulation of double-strand break repair via homologous recombination	Chek1	0.007033519
GO:0016601	Rac protein signal transduction	Eps8l1	0.009368012
GO:0031572	G2 DNA damage checkpoint	Chek1	0.009834312
	**Cellular Component**		
GO:0070382	exocytic vesicle	Sytl2	0.005664978
GO:0005657	replication fork	Chek1	0.009425405
GO:0019897	extrinsic component of plasma membrane	Sytl2	0.012705168
GO:0042470	melanosome	Sytl2	0.012705168
GO:0030863	cortical cytoskeleton	Mpp1	0.015041809
GO:0000794	condensed nuclear chromosome	Chek1	0.019235097
GO:0000781	chromosome, telomeric region	Chek1	0.023875266
GO:0032587	ruffle membrane	Eps8l1	0.02526342
GO:0001726	ruffle	Eps8l1	0.034013467
GO:0005604	basement membrane	Papln	0.04587151
	**Molecular Function**		
GO:0004629	phospholipase C activity	Plcb2	0.004805373
GO:0042043	neurexin family protein binding	Sytl2	0.006242886
GO:0030676	Rac guanyl-nucleotide exchange factor activity	Eps8l1	0.006721637
GO:0042608	T cell receptor binding	Eps8l1	0.007678511
GO:0004435	phosphatidylinositol phospholipase C activity	Plcb2	0.008634546
GO:0003779	actin binding	Eps8l1;Tns4	0.010618993
GO:0001786	phosphatidylserine binding	Sytl2	0.011497631
GO:0031683	G-protein beta/gamma-subunit complex binding	Plcb2	0.01197408
GO:0008081	phosphoric diester hydrolase activity	Plcb2	0.02240329
GO:0005544	calcium-dependent phospholipid binding	Sytl2	0.02240329

Supplementary Table 11. The top 10 enriched GO terms of DEGs between the KE and KO groups.

ID	GO term	Enriched genes	*P* value
	**Biological Process**		
GO:0071493	cellular response to UV-B	Hyal2;Cdkn1a	8.09E-05
GO:0006977	DNA damage response	Cdkn1a;Pidd1	0.000118457
GO:0043066	negative regulation of apoptotic process	Mdm4;Bcl11b;Pidd1;Hspa1b;Cdkn1a	0.000407
GO:2001238	positive regulation of extrinsic apoptotic signaling pathway	Hyal2;Pidd1	0.000988155
GO:0055085	transmembrane transport	Itpr2;Oca2;Slc16a8;Kcnq5	0.002144141
GO:0032482	Rab protein signal transduction	Rab17;Rab19	0.003786561
GO:0006886	intracellular protein transport	Rab17;Rab19;Sytl2	0.004540113
GO:0006547	histidine metabolic process	Ftcd	0.008174517
GO:0033153	T cell receptor V(D)J recombination	Bcl11b	0.008174517
GO:0044027	hypermethylation of CpG island	Kcnq1ot1	0.008174517
	**Cellular Component**		
GO:0042470	melanosome	Rab17;Sytl2	0.000635581
GO:0005622	intracellular	Bcl11b;Hspa1b;Eps8l1;Slfn9; Cdkn1a;Rab17;Ift80	0.001358343
GO:0030139	endocytic vesicle	Hyal2;Rab17	0.002529989
GO:0001726	ruffle	Eps8l1;Frmd4b	0.004371725
GO:0005886	plasma membrane	D930015E06Rik;Hyal2;Veph1;Lima1; Itpr2;Slc16a8;Glra3;Sytl2;Rab17;Rab19	0.010363304
GO:0097458	neuron part	Bcl11b	0.010611343
GO:0000139	Golgi membrane	Hyal2;Ftcd	0.01112583
GO:0000307	cyclin-dependent protein kinase holoenzyme complex	Cdkn1a	0.013247104
GO:0030686	90S preribosome	Gm6252	0.013247104
GO:0070382	exocytic vesicle	Sytl2	0.01587607
	**Molecular Function**		
GO:0016934	extracellular-glycine-gated chloride channel activity	Glra3	0.007847372
GO:0015129	lactate transmembrane transporter activity	Slc16a8	0.009149467
GO:0051015	actin filament binding	Lima1;Eps8l1	0.010284424
GO:0015278	calcium-release channel activity	Itpr2	0.010449909
GO:0019912	cyclin-dependent protein kinase activating kinase activity	Cdkn1a	0.011748702
GO:0004415	hyalurononglucosaminidase activity	Hyal2	0.013045847
GO:0008028	monocarboxylic acid transmembrane transporter activity	Slc16a8	0.0156352
GO:0042043	neurexin family protein binding	Sytl2	0.016927413
GO:0004861	cyclin-dependent protein serine/ threonine kinase inhibitor activity	Cdkn1a	0.016927413
GO:0070679	inositol 1,4,5 trisphosphate binding	Itpr2	0.016927413

Supplementary Table 12. The top 10 enriched GO terms of DEGs between the WE and WT groups.

ID	GO term	Enriched genes	*P* value
	**Biological Process**		
GO:0006900	membrane budding	Trim72;Wasl;Vps4a	0.000156763
GO:0051056	regulation of small GTPase mediated signal transduction	Iqsec3;Rap1gap2;Cdc42bpa	0.0011777
GO:0070588	calcium ion transmembrane transport	Atp2c1;Oprm1;Loxhd1;Cacna1h;Ryr1;Trpm1;Slc8b1	0.001319117
GO:0071320	cellular response to cAMP	Igfbp5;Akap7;Egr3;Hcn1	0.001573349
GO:0015914	phospholipid transport	Pitpnb;Atp11a;Pctp;Tmem30a	0.002442009
GO:2000344	positive regulation of acrosome reaction	Cacna1h;Plb1	0.003180204
GO:0070863	positive regulation of protein exit from endoplasmic reticulum	Slc51b;Tmem30a	0.003180204
GO:0045759	negative regulation of action potential	Cnr2;Hcn1	0.003180204
GO:0018119	peptidyl-cysteine S-nitrosylation	S100a9;S100a8	0.003180204
GO:0009804	coumarin metabolic process	Cyp2a5;Cyp2a4	0.003180204
	**Cellular Component**		
GO:0042383	sarcolemma	Oprm1;Trim72;Cacna1h;Ryr1;Col6a2;Slc8b1	0.003030434
GO:0016023	cytoplasmic membrane-bounded vesicle	Hyal2;Sgk3;Axin2;Wasl;Chil3	0.0046114
GO:0090543	Flemming body	Vps4a;Ist1	0.005601224
GO:0000139	Golgi membrane	Hyal2;Atp2c1;Xylt1;Wasl;Copg1	0.021606423
GO:0005881	cytoplasmic microtubule	Cyp2a4;Cyp2a5;Axin2	0.030840161
GO:0030992	intraciliary transport particle B	Rabl2;Ttc30a2	0.032204495
GO:0005871	kinesin complex	Kif5b;Kif12;Kif1c	0.033902314
GO:0016607	nuclear speck	Apex1;Dyrk1a;Ppih;1810011O10Rik	0.034440442
GO:0030139	endocytic vesicle	Rabep1;Kif5b;Hyal2	0.03711623
GO:0016529	sarcoplasmic reticulum	Ryr1;Akap7;Syne2	0.038779671
	**Molecular Function**		
GO:0035662	Toll-like receptor 4 binding	S100a9;S100a8	0.003349627
GO:0016798	hydrolase activity, acting on glycosyl bonds	Hyal2;Lyz1;Lyzl6;Tdg;Chil3	0.004014281
GO:0008536	Ran GTPase binding	Kpnb1;Xpo6;Rangrf	0.005512792
GO:0043425	bHLH transcription factor binding	Usf2;Ep300;Tcf4	0.006090798
GO:0050786	RAGE receptor binding	S100a9;S100a8	0.006996174
GO:0050544	arachidonic acid binding	S100a9;S100a8	0.006996174
GO:0008565	protein transporter activity	Ap2b1;Kpnb1;Xpo6;Rangrf	0.008477855
GO:0003796	lysozyme activity	Lyz1;Lyzl6	0.008478305
GO:0008017	microtubule binding	Kif1c;S100a9;Kif5b;Ppp5c;S100a8;Ska2;Kif12	0.009382088
GO:0019904	protein domain specific binding	Vps4a;E2f4;Oprm1;Ist1;Akap7;Kpnb1;Stx18;Ppp1r2;Tdg	0.011967994

Supplementary Table 13. The top 10 enriched GO terms of DEGs between the KE and WT groups.

ID	GO term	Enriched genes	*P* value
	**Biological Process**		
GO:0048844	artery morphogenesis	Prrx1;Smad7;Nf1;Stra6;Hes1	0.000102012
GO:0051056	regulation of small GTPase mediated signal transduction	Cdc42bpa;Rap1gap2;Iqsec3	0.002302552
GO:0030325	adrenal gland development	Stra6;Nf1;Pdgfra	0.004916793
GO:0072711	cellular response to hydroxyurea	Rad51;Atrx	0.005029769
GO:0070863	positive regulation of protein exit from endoplasmic reticulum	Tmem30a;Slc51b	0.005029769
GO:0045759	negative regulation of action potential	Cnr2;Hcn1	0.005029769
GO:0065004	protein-DNA complex assembly	Tcf4;Ep300	0.006636052
GO:0006900	membrane budding	Wasl;Vps4a	0.006636052
GO:2000323	negative regulation of glucocorticoid receptor signaling pathway	Phb;Cry1	0.006636052
GO:0017148	negative regulation of translation	Paip2b;Tia1;Gigyf2;Trim71;Igfbp5	0.006900816
	**Cellular Component**		
GO:0031527	filopodium membrane	Antxr1;Palm;Syne2	0.002901969
GO:0031258	lamellipodium membrane	Antxr1;Syne2	0.006918742
GO:0005793	endoplasmic reticulum-Golgi intermediate compartment	Ptpn2;Rgmb;Nucb1;Ist1	0.008793303
GO:0090543	Flemming body	Vps4a;Ist1	0.008800374
GO:0000139	Golgi membrane	Xylt1;Hyal2;Copg1;Wasl;<br>Arfip1;Atp2c1	0.015973912
GO:0035102	PRC1 complex	Phc2;Cbx8	0.01826734
GO:0000792	heterochromatin	Phc2;Cbx8;Atrx	0.020481389
GO:0005798	Golgi-associated vesicle	Map6d1;Nucb1	0.02108549
GO:0005902	microvillus	Lyz1;Hyal2;Spn;Pdgfra	0.023635841
GO:0016363	nuclear matrix	Zfp326;Scaf8;Runx1t1;Hes1	0.041483935
	**Molecular Function**		
GO:0015026	coreceptor activity	Gfra4;Rgmb;Cd22	0.003419784
GO:0019904	protein domain specific binding	E2f4;Tdg;Ist1;Kpnb1;Akap7;Scaf8;<br>Ppp1r2;Homer2;Stx18;Vps4a;Spn	0.007541022
GO:0045545	syndecan binding	Sema5a;Nf1	0.008827046
GO:0008022	protein C-terminus binding	Phb;Tcf4;Akap7;Ksr1;Pdzd11;<br>Rad51;Ep300;Vps4a;Hcn1	0.009739438
GO:0008536	Ran GTPase binding	Xpo6;Rangrf;Kpnb1	0.010378605
GO:0043425	bHLH transcription factor binding	Usf2;Tcf4;Ep300	0.011438707
GO:0031490	chromatin DNA binding	Apex1;Prdm14;Vax2;Ep300	0.011540636
GO:0005515	protein binding	Rab3d;Ksr1;Smad7;Gkap1;Ist1;Kcnq<br>1ot1;Runx1t1;Sim1;Ptpn2;Cbx1;etc	0.013121392
GO:0004629	phospholipase C activity	Plcb2;Plcd4	0.01319896
GO:0032403	protein complex binding	Mb21d2;Casp3;Kpnb1;Ist1;Otof;Pdg<br>fra;Ppfia2;Ins2;Ep300;Apex1;etc	0.014087821

Supplementary Table 14. The top 10 enriched GO terms of DEGs between the KE and WE groups.

ID	GO term	Enriched genes	*P* value
	**Biological Process**		
GO:0031077	post-embryonic camera-type eye development	Bcl11b	0.007665899
GO:0010574	regulation of vascular endothelial growth factor production	Ccr2	0.007665899
GO:0010837	regulation of keratinocyte proliferation	Bcl11b	0.007665899
GO:0019725	cellular homeostasis	Ccr2	0.007665899
GO:0033153	T cell receptor V(D)J recombination	Bcl11b	0.007665899
GO:0006003	fructose 2,6-bisphosphate metabolic process	Gck	0.007665899
GO:0031584	activation of phospholipase D activity	Hpca	0.008938013
GO:0002829	negative regulation of type 2 immune response	Ccr2	0.008938013
GO:0032811	negative regulation of epinephrine secretion	Gck	0.008938013
GO:0045627	positive regulation of T-helper 1 cell differentiation	Ccr2	0.008938013
	**Cellular Component**		
GO:0008250	oligosaccharyltransferase complex	Tusc3	0.008992746
GO:0097458	neuron part	Bcl11b	0.010271025
GO:0045180	basal cortex	Gck	0.010271025
GO:0044327	dendritic spine head	Hpca	0.012822804
GO:0005765	lysosomal membrane	Tmem192;Oca2	0.018663317
GO:0032839	dendrite cytoplasm	Hpca	0.028000441
GO:0005768	endosome	Tmem192;Rab17; Zfyve28	0.02954089
GO:0042470	melanosome	Rab17	0.03425766
GO:0032590	dendrite membrane	Hpca	0.03425766
GO:0032809	neuronal cell body membrane	Hpca	0.03425766
	**Molecular Function**		
GO:0004396	hexokinase activity	Gck	0.007065876
GO:0004340	glucokinase activity	Gck	0.007065876
GO:0005536	glucose binding	Gck	0.01524864
GO:0016493	C-C chemokine receptor activity	Ccr2	0.01757462
GO:0015095	magnesium ion transmembrane transporter activity	Tusc3	0.01757462
GO:0031210	phosphatidylcholine binding	Pctp	0.019895309
GO:0019955	cytokine binding	Ccr2	0.023366447
GO:0005540	hyaluronic acid binding	Lyve1	0.027976222
GO:0004950	chemokine receptor activity	Ccr2	0.029125387
GO:0032266	phosphatidylinositol-3-phosphate binding	Zfyve28	0.03370898

Supplementary Table 15. Common KEGG terms emerged in both comparisons.

ID	KEGG term	Enriched genes (KO VS WT)	Enriched genes (KE VS KO)
**Up-regulated pathway**			
mmu04115	p53 signaling pathway	Chek1	Cdkn1a, Mdm4, Pidd1
**Down-regulated pathway**			
mmu04730	Long-term depression	Plcb2	Itpr2
mmu04915	Estrogen signaling pathway	Plcb2	Itpr2, Hspa1b

Supplementary Table 16. The top 20 enriched KEGG terms of DEGs between the KO and WT groups.

ID	KEGG term	Count	PopHit	Enriched	*P* value
mmu05143	African trypanosomiasis	1	36	Plcb2	0.009128992
mmu04973	Carbohydrate digestion and absorption	1	43	Plcb2	0.01085139
mmu04961	Endocrine and other factor-regulated calcium reabsorption	1	54	Plcb2	0.013554988
mmu04730	Long-term depression	1	61	Plcb2	0.015273533
mmu04115	p53 signaling pathway	1	67	Chek1	0.016745379
mmu04720	Long-term potentiation	1	67	Plcb2	0.016745379
mmu04924	Renin secretion	1	72	Plcb2	0.017971077
mmu00562	Inositol phosphate metabolism	1	72	Plcb2	0.017971077
mmu04918	Thyroid hormone synthesis	1	73	Plcb2	0.018216124
mmu04971	Gastric acid secretion	1	74	Plcb2	0.018461142
mmu04970	Salivary secretion	1	78	Plcb2	0.019440905
mmu04911	Insulin secretion	1	85	Plcb2	0.021154313
mmu04540	Gap junction	1	86	Plcb2	0.021398963
mmu04925	Aldosterone synthesis and secretion	1	86	Plcb2	0.021398963
mmu04912	GnRH signaling pathway	1	89	Plcb2	0.02213273
mmu04915	Estrogen signaling pathway	1	96	Plcb2	0.023843784
mmu04713	Circadian entrainment	1	98	Plcb2	0.024332381
mmu04070	Phosphatidylinositol signaling system	1	98	Plcb2	0.024332381
mmu04916	Melanogenesis	1	100	Plcb2	0.024820856
mmu04933	AGE-RAGE signaling pathway in diabetic complications	1	100	Plcb2	0.024820856

Note：Count, the number of genes enriched in specific KEGG terms; PopHit, Population Hit, the number of genes annotated in specific KEGG terms.

Supplementary Table 17. The top 20 enriched KEGG terms of DEGs between the KE and KO groups.

ID	KEGG term	Count	PopHit	Enriched genes	*P* value
mmu04115	p53 signaling pathway	3	67	Cdkn1a;Mdm4;Pidd1	7.11E-05
mmu04210	Apoptosis	3	136	Itpr2;Pidd1;Bcl2a1c	0.000544095
mmu04915	Estrogen signaling pathway	2	96	Itpr2;Hspa1b	0.006126744
mmu04064	NF-kappa B signaling pathway	2	104	Pidd1;Bcl2a1c	0.007135674
mmu04725	Cholinergic synapse	2	113	Itpr2;Kcnq5	0.008355333
mmu04921	Oxytocin signaling pathway	2	153	Cdkn1a;Itpr2	0.014816667
mmu05202	Transcriptional misregulation in cancer	2	177	Cdkn1a;Bcl2a1c	0.019466689
mmu04218	Cellular senescence	2	187	Cdkn1a;Itpr2	0.021566129
mmu00670	One carbon pool by folate	1	19	Ftcd	0.024444178
mmu05167	Kaposi's sarcoma-associated herpesvirus infection	2	204	Cdkn1a;Itpr2	0.025345465
mmu05205	Proteoglycans in cancer	2	206	Cdkn1a;Itpr2	0.025807118
mmu00531	Glycosaminoglycan degradation	1	21	Hyal2	0.02685877
mmu05169	Epstein-Barr virus infection	2	220	Cdkn1a;Hspa1b	0.029136503
mmu00340	Histidine metabolism	1	25	Ftcd	0.031671824
mmu05206	MicroRNAs in cancer	2	281	Cdkn1a;Mdm4	0.045523727
mmu05219	Bladder cancer	1	41	Cdkn1a	0.050710538
mmu05134	Legionellosis	1	58	Hspa1b	0.070569428
mmu04730	Long-term depression	1	61	Itpr2	0.074034863
mmu04213	Longevity regulating pathway-multiple species	1	62	Hspa1b	0.075187421
mmu05214	Glioma	1	64	Cdkn1a	0.077488665

Note：Count, the number of genes enriched in specific KEGG terms; PopHit, Population Hit, the number of genes annotated in specific KEGG terms.

Supplementary Table 18. The top 20 enriched KEGG terms of DEGs between the WE and WT groups.

ID	KEGG term	Count	PopHit	Enriched genes	*P* value
mmu04144	Endocytosis	10	286	Vps4a;Gm8909;Kif5b;Ist1;Iqsec3; H2-Q1;Rabep1;Eea1;Wasl;Ap2b1	0.010638002
mmu03410	Base excision repair	3	35	Apex1;Hmgb1;Tdg	0.017352165
mmu05203	Viral carcinogenesis	8	231	Gm8909;Hist1h2bb;Ep300;Pik3cd ;H2-Q1;Casp3;Ranbp1;Egr3	0.022632799
mmu03013	RNA transport	6	167	Gm9839;AF366264;Kpnb1; Upf3b;Eif3j2;Eif1	0.039646722
mmu00515	Mannose type O-glycan biosynthesis	2	23	Large;B3galnt2	0.050373382
mmu04977	Vitamin digestion and absorption	2	24	Lrat;Plb1	0.054065857
mmu00563	Glycosylphosphatidylinositol (GPI)-anchor biosynthesis	2	25	Pigb;Pigg	0.057851097
mmu04950	Maturity onset diabetes of the young	2	27	Hes1;Ins2	0.065686286
mmu04922	Glucagon signaling pathway	4	102	Phka1;Ep300;Gcgr;Plcb2	0.067782835
mmu05210	Colorectal cancer	3	62	Casp3;Axin2;Pik3cd	0.067995381
mmu04310	Wnt signaling pathway	5	146	Gm9839;Plcb2;Ep300;Axin2; AF366264	0.068196654
mmu05016	Huntington's disease	6	194	Cox7c;Plcb2;Dnal1;Ep300; Casp3;Ap2b1	0.070358373
mmu05146	Amoebiasis	4	106	Plcb2;Casp3;Serpinb6c;Pik3cd	0.075455761
mmu04932	Non-alcoholic fatty liver disease (NAFLD)	5	151	Cox7c;Mlx;Pik3cd;Ins2;Casp3	0.076084982
mmu04931	Insulin resistance	4	109	Mlx;Pik3cd;Ins2;Ppp1r3b	0.081487103
mmu04940	Type I diabetes mellitus	3	70	H2-Q1;Ins2;Gm8909	0.089243539
mmu04520	Adherens junction	3	72	Wasl;Ep300;Pvrl4	0.094944201
mmu05100	Bacterial invasion of epithelial cells	3	76	Elmo1;Wasl;Pik3cd	0.106779222
mmu04960	Aldosterone-regulated sodium reabsorption	2	38	Pik3cd;Ins2	0.114048583
mmu04068	FoxO signaling pathway	4	132	Ins2;Ep300;Pik3cd;Sgk3	0.135038658

Note：Count, the number of genes enriched in specific KEGG terms; PopHit, Population Hit, the number of genes annotated in specific KEGG terms.

Supplementary Table 19. The top 20 enriched KEGG terms of DEGs between the KE and WT groups.

ID	KEGG term	Count	PopHit	Enriched genes	*P* value
mmu04144	Endocytosis	12	286	Eea1;Erbb4;Ist1;Pdgfra;Gm8909; H2-Q1;Kif5b;Rabep1;Ap2b1; Wasl;Vps4a;Iqsec3	0.005018707
mmu00563	Glycosylphosphatidylinositol (GPI)-anchor biosynthesis	3	25	Pigg;Pigb;Pign	0.011800954
mmu00640	Propanoate metabolism	3	31	Pcca;Dbt;Abat	0.020011716
mmu05016	Huntington's disease	8	194	Casp3;Polr2l;Plcb2;Ap2b1;Ep300; Cox7c;Sdha;Dnal1	0.022609904
mmu03410	Base excision repair	3	35	Tdg;Apex1;Hmgb1	0.026853535
mmu04066	HIF-1 signaling pathway	5	105	Pik3cd;Ins2;Ep300;Pgk1;Rps6kb1	0.040315982
mmu04931	Insulin resistance	5	109	Ppp1r3b;Pik3cd;Ins2;Mlx;Rps6kb1	0.045846837
mmu04932	Non-alcoholic fatty liver disease (NAFLD)	6	151	Casp3;Pik3cd;Ins2;Mlx;Cox7c;Sdha	0.052647317
mmu01521	EGFR tyrosine kinase inhibitor resistance	4	80	Pik3cd;Nf1;Rps6kb1;Pdgfra	0.05576429
mmu04630	Jak-STAT signaling pathway	6	159	Pik3cd;Ifna14;Ptpn2;Ep300; Il21r;Il22ra1	0.06394295
mmu04350	TGF-beta signaling pathway	4	85	E2f4;Smad7;Rps6kb1;Ep300	0.066184875
mmu04666	Fc gamma R-mediated phagocytosis	4	87	Wasl;Limk1;Rps6kb1;Pik3cd	0.070618462
mmu05215	Prostate cancer	4	87	Pik3cd;Ins2;Pdgfra;Ep300	0.070618462
mmu05221	Acute myeloid leukemia	3	55	Pik3cd;Runx1t1;Rps6kb1	0.076950579
mmu04621	NOD-like receptor signaling pathway	6	168	Gm5136;Ifna14;Irak4;Plcb2; Naip7;Antxr1	0.078236332
mmu04514	Cell adhesion molecules (CAMs)	6	169	Cd276;Gm8909;H2-Q1;Icosl; Spn;Cd22	0.079927829
mmu00280	Valine, leucine and isoleucine degradation	3	56	Pcca;Dbt;Abat	0.080099124
mmu04068	FoxO signaling pathway	5	132	Pik3cd;Sgk3;Homer2;Ins2;Ep300	0.086039782
mmu04950	Maturity onset diabetes of the young	2	27	Ins2;Hes1	0.087690663
mmu05169	Epstein-Barr virus infection	7	220	Pik3cd;Polr2l;Gm8909;H2-Q1; Ep300;Spn;Adrm1	0.094860112

Note：Count, the number of genes enriched in specific KEGG terms; PopHit, Population Hit, the number of genes annotated in specific KEGG terms.

Supplementary Table 20. The top 20 enriched KEGG terms of DEGs between the KE and WE groups.

ID	KEGG term	Count	PopHit	Enriched genes	*P* value
mmu00524	Neomycin, kanamycin and gentamicin biosynthesis	1	5	Gck	0.003703243
mmu00400	Phenylalanine, tyrosine and tryptophan biosynthesis	1	8	Lao1	0.005550745
mmu00360	Phenylalanine metabolism	1	23	Lao1	0.014747192
mmu04950	Maturity onset diabetes of the young	1	27	Gck	0.01718805
mmu00052	Galactose metabolism	1	32	Gck	0.020232317
mmu00500	Starch and sucrose metabolism	1	33	Gck	0.020840264
mmu00250	Alanine, aspartate and glutamate metabolism	1	37	Lao1	0.023269036
mmu00350	Tyrosine metabolism	1	39	Lao1	0.024481613
mmu00380	Tryptophan metabolism	1	46	Lao1	0.028716153
mmu00270	Cysteine and methionine metabolism	1	48	Lao1	0.029923316
mmu04930	Type II diabetes mellitus	1	48	Gck	0.029923316
mmu00510	N-Glycan biosynthesis	1	49	Tusc3	0.030526448
mmu00520	Amino sugar and nucleotide sugar metabolism	1	49	Gck	0.030526448
mmu01100	Metabolic pathways	3	1316	Gck;Lao1;Tusc3	0.03337417
mmu00280	Valine, leucine and isoleucine degradation	1	56	Lao1	0.034739974
mmu05230	Central carbon metabolism in cancer	1	64	Gck	0.039537479
mmu00010	Glycolysis / Gluconeogenesis	1	66	Gck	0.04073387
mmu04917	Prolactin signaling pathway	1	72	Gck	0.04431589
mmu04911	Insulin secretion	1	85	Gck	0.052040231
mmu04922	Glucagon signaling pathway	1	102	Gck	0.062065844

Note：Count, the number of genes enriched in specific KEGG terms; PopHit, Population Hit, the number of genes annotated in specific KEGG terms.
